# Assessment of Personality in Basque Public Sector Employees and Its Role in Predicting Organizational Citizenship Behaviors in Selection Processes

**DOI:** 10.3389/fpsyg.2021.787850

**Published:** 2021-12-10

**Authors:** Nekane Balluerka, Arantxa Gorostiaga, Alexander Rodríguez-López, Jone Aliri

**Affiliations:** ^1^Department of Clinical and Health Psychology and Research Methods, Faculty of Psychology, University of the Basque Country (UPV/EHU), San Sebastian, Spain; ^2^Provincial Council of Alava, Vitoria-Gasteiz, Spain

**Keywords:** test adaptation, personality, organizational citizenship behaviors, personnel selection, public sector

## Abstract

Organizational citizenship behaviors (OCBs) are an important aspect of job performance as they enhance the effectiveness of organizations. Research has shown that personality is a moderate predictor of job performance. This study, involving a sample of 678 public sector employees in the Basque Country (northern Spain), pursued two aims: First, to develop and validate a Basque-language version of the Overall Personality Assessment Scale (OPERAS), a scale designed to assess the Big Five personality factors in a wide range of settings; and second, to examine whether person-organization fit (PO fit) and adaptive performance improve the capacity of personality to predict OCBs. The results indicated that the adapted scale was a suitable instrument for assessing personality in the Basque-speaking population. Furthermore, PO fit and adaptive performance improved the capacity of personality to predict OCBs. Based on these results, we propose a new predictive model that may enhance the efficiency of personnel selection processes.

## Introduction

Organizational citizenship behaviors (OCBs) are an important aspect of job performance as they enhance the effectiveness and management of organizations (Podsakoff et al., 2009; Organ, 2018). These behaviors are considered an example of extra-role performance, in contrast to the in-role behaviors or task performance that are required by the person’s job description. Accordingly, OCBs have been defined as “individual contributions in the workplace that go beyond role requirements and contractually rewarded job achievements” (Organ and Ryan, 1995, p. 775).

The meta-analysis by Chiaburu et al. (2017) concluded that overall job performance was determined relatively more by OCBs than by task performance. This finding is consistent with the results of a study by Lievens et al. (2008), who found that more weight was given to OCBs in team-based organizational cultures, and also by peers as opposed to supervisors. The greater relative weight of OCBs may be due to the growing attention that has been paid to aspects of extra-role performance over the past decade or so (Carpini and Parker, 2017), reflecting an important conceptual shift away from the emphasis in previous research on task performance (Conway, 1999; Rotundo and Sackett, 2002).

Although there are a variety of conceptual models of OCB and its different dimensions, the most widely used framework among researchers in the field (Ilies et al., 2009; Chiaburu et al., 2011, 2017; Organ, 2018) is that described by Williams and Anderson (1991), who categorized the five main types of behavior (altruism, courtesy, conscientiousness, civic virtue, and sportsmanship) proposed by Organ (1988) into two broad groups according to the intended target: *OCBs directed at the organization* (*OCB-Os*), behaviors that target the organization in order to improve its functioning, and *OCBs directed at individuals* (*OCB-Is*), behaviors directed at specific people within the organization to assist them with their work or personal problems (Dávila and Finkelstein, 2010). This model is similar to the classification of contextual performance described by Borman and Motowidlo (1993), who distinguish between three sub-dimensions: *Personal support*, which has similarities to OCB-I; *Organizational support*, similar to OCB-O; and what they refer to as *Conscientiousness initiative* (Dorsey et al., 2010).

The goal of personnel selection is to identify those candidates most likely to perform well at work, that is, those whose behavior will meet or exceed the standards set by the organization (Barrick et al., 2011; Hughes and Batey, 2017). Understanding the variables that predict job performance is therefore particularly important for personnel selection. One of the most influential papers in the history of work and organizational psychology, the meta-analysis by Barrick and Mount (1991), highlighted the decisive role that personality plays in job performance, and more recent studies (Shaffer and Postlethwaite, 2012; Judge et al., 2013; Wilmot and Ones, 2019; Soto, 2021) have confirmed this relationship focusing on the Big Five model. This model posits that personality traits can be organized in terms of five broad domains: Extraversion (a person’s level of sociability, including characteristics such as talkativeness, preference for animated social situations), Agreeableness (the tendency to be friendly and consider others’ feelings and rights, including characteristics such as empathy, cooperation, honesty or trust in others), Conscientiousness (a person’s degree of responsibility, including characteristics such as planning, organization and efficiency), Emotional Stability (tendency to feel calm, and a low propensity to feel negative emotions such as anxiety, insecurity, sadness or fear), and Openness to Experience (a person’s disposition to consider other ways of thinking and the interest in living new experiences, including characteristics such as imagination, curiosity, interest in culture and art, etc.).

Furthermore, although personality traits are at best only moderate predictors of job performance (Morgeson et al., 2007), they are considered important because personality usually shows low correlations with variables that are commonly assessed as part of selection tests, such as intelligence or job knowledge; hence, personality may be a crucial variable to consider when choosing the best candidates. In addition, and as shown in the meta-analysis by Ones et al. (2007), personality dimensions not only predict job performance but are also good predictors of other important aspects of organizations such as teamwork, counterproductive behaviors, customer service, job satisfaction, commitment to the organization, leadership or training success.

Moreover, personality is related to public sector motivation and ethical conduct, which are very relevant issues in the public sphere (Van Witteloostuijn et al., 2017; Wiernik and Ones, 2018). Another characteristic that affects personnel selection in the public sector is that it involves taking into account the legal context and its implications for practice. Thus, in Spain it is necessary to consider both the constitutional rights of citizens and the Basic Statute of the Public Employee. The Basic Statute, in particular, requires the selection of public employees to identify competencies and personal characteristics related to effective job performance. Therefore, personality assessment could contribute to improve selection systems in the public sector (Salgado and Moscoso, 2008; Shen et al., 2017).

The results of meta-analyses generally suggest that the relationship between personality and behavior is stronger when performance is more discretionary and volitional, as in the case of contextual performance, than it is with behaviors that are more closely monitored and structured, as in task performance (Chiaburu et al., 2011; Barrick and Mount, 2014). Accordingly, personality tests are especially useful for predicting “will-do” behaviors that are associated with motivation and with the level of attention, direction, intensity, and persistence of effort that an employee is willing to exert in a given situation, as opposed to “can-do” behaviors that are influenced by aptitude and skill; they also assess traits that reflect whether an individual is likely to follow organizational rules or norms (Campbell, 1990; Barrick and Mount, 2014). Consequently, if the aim is to predict aspects of extra-role performance such as OCBs, which are fundamentally influenced by people’s motivation, then personality would seem to be an essential construct to consider during the selection process. A number of meta-analyses have examined in depth the relationship between personality and OCBs. Specifically, Chiaburu et al. (2011) found that Conscientiousness, Agreeableness, and Openness to Experience were the personality dimensions most strongly correlated with OCBs, while Judge et al. (2013) concluded that Conscientiousness, Extraversion, and Agreeableness were the dimensions that best predicted OCBs (Sackett and Walmsley, 2014). For their part, Ilies et al. (2009) found that Agreeableness was more closely related to OCB-Is, whereas Conscientiousness showed a higher correlation with OCB-Os.

Given the possible role of personality in the prediction of OCBs and therefore in selection processes, it needs to be carefully evaluated. This evaluation requires using an instrument that offers good psychometric properties and is well adapted to the cultural context in which it is used. Thus, given that the setting for our research is a bilingual region of northern Spain in which Basque is the first language of 20.5% of the population (Basque Government, 2019), the primary aim of our study was to develop a Basque-language version of the Overall Personality Assessment Scale (OPERAS) (Vigil-Colet et al., 2013), an instrument based on the Big Five model of personality. Although Basque legislation calls for guaranteeing the linguistic rights of citizens in civil service exams and competitions, it is essential to adapt more assessment measures to Basque because to date, the only personality measures available in Basque are adaptations of the NEO-PI and NEO-FFI (Balluerka et al., 2007; Gorostiaga et al., 2011). In comparison with these scales, the OPERAS offers a number of advantages: It has excellent psychometric properties (Vigil-Colet et al., 2013); its design fulfills the recommendations of Soto and John (2019) for increasing the external validity of measurement instruments (i.e., scales of between six and nine items which measure broad factors and are balanced in terms of positively and negatively worded items); it is relatively brief (40 items) and is easy to complete. It also incorporates an innovative procedure for controlling acquiescence and social desirability, the two main response biases (Paulhus, 1991), which may both be present simultaneously (Hofstee et al., 1998). A further advantage of the scale is the fact that its items do not refer specifically to the employment context; since the responses do not depend on a candidate’s level of work experience, it is a more equitable measure. For these reasons we considered the OPERAS to be a highly suitable test for assessing personality in selection processes and decided to select this instrument for adaptation to Basque.

As noted on the research reviewed above, we may conclude that personality is a relevant predictor of job performance, assessed in terms of OCBs, although its predictive capacity is only moderate. Consequently, other variables also need to be taken into account in order to improve the effectiveness of selection processes. The I-ADAPT theoretical model of Ployhart and Bliese (2006) provides a framework for identifying other variables that may enhance the predictive capacity of personality. The I-ADAPT model proposes that knowledge, skills, values, aptitudes, and personality predict adaptive performance, defined as the ability to adapt successfully to changing tasks, and that this, in turn, predicts task and contextual performance; this has been partially confirmed by various studies that have analyzed the relationship between person-organization fit (PO fit) and adaptive performance, or between adaptive performance and contextual performance (Chan and Schmitt, 2002; Wang et al., 2011). Application of the I-ADAPT model to the context of personnel selection leads us to speculate that PO fit and adaptive performance may be predictors of OCBs. In this respect, the meta-analysis by Hoffman and Woehr (2006) found a correlation of moderate magnitude between PO fit and OCBs overall (without distinguishing between OCB-O and OCB-I), while Charbonnier-Voirin and Roussel (2012) reported a high correlation between an overall measure of adaptive performance and OCBs. According to Murphy (2015), these relationships may be explained by the fact that both constructs include competencies for monitoring and assessing a situation and using that information to effectively adjust behavior.

In light of the above, the second aim of the present study was to examine whether personality predicts OCBs, and if so, whether its predictive capacity is improved by also taking into account PO fit and adaptive performance. In this regard, it should be noted that Tsai et al. (2012), in a group of high-tech employees, and Michaud (2014), in a sample of public sector workers, found that PO fit enhanced the capacity of personality to predict OCBs. However, their samples were small and they obtained different effect sizes (Δ*R*^2^ = 0.02 and Δ*R*^2^ = 0.33, respectively). To the best of our knowledge, adaptive performance has yet to be added to a predictive model of this kind. Based on the I-ADAPT model and the relationship that both PO fit and adaptive performance show with OCBs, we hypothesize that the capacity of personality to predict OCBs will be notably increased by including these two variables in the model.

In summary, our first objective was to develop a valid and reliable instrument for assessing personality in the Basque Country. We also aimed to propose a new and efficient predictive model for selecting the candidates most likely to perform well at work through the administration of short, valid instruments. The effectiveness of the proposed model would help to provide evidence of validity based on relations to other variables, for the Basque adaptation of the instrument.

## Materials and Methods

### Participants

To recruit the participants, the collaboration of the 62 largest Basque public entities was requested, with a total of 32 organizations agreeing to participate in the study. Criteria for inclusion were being a public employee and having a medium-high level of Basque (B1 to C2 levels in the Common European Framework of Reference for Languages).

The sample for this study comprised 678 public sector employees (444 women), with a mean age of 44.63 years (*SD* = 7.66). They had been working for an average of 11.46 years (SD = 7.66) in local and regional government organizations in the Basque Country, in sectors such as health (10%), education (12%), police and security (8%), public finances (15%), human resources (19%), work and pensions (9%), and environmental protection (10%). Regarding their level of education, 420 had a degree, 120 a Diploma of Higher Education, 113 had completed high school, and 25 had only primary-level education. Of those with a degree or diploma, 384 were in posts that required a university qualification, which was not necessary for the remainder. In the case of 213 participants, their job included responsibility for making decisions about the work of other employees.

### Instruments

*Overall Personality Assessment Scale (OPERAS; Vigil-Colet et al., 2013; in its Basque version, developed for the present study).* The OPERAS is a 40-item scale that assesses the Big Five personality traits (Agreeableness, Openness to Experience, Emotional Stability, Extraversion, and Conscientiousness), and it is applicable to a wide range of settings: clinical, human resources, education, research, etc. Four of its items are designed to capture social desirability and one (item 1) is a dummy item which the scale authors included as a training item. The remaining 35 items are distributed evenly across the five personality factors. All items are rated on a 5-point Likert-type scale (from 1, “fully disagree,” to 5, “fully agree”). In the original validation study (Vigil-Colet et al., 2013), the reliability estimates (internal consistency of factor scores) were above 0.70 (Agreeableness 0.71; Openness to Experience 0.81; Emotional Stability 0.86; Extraversion 0.86; and Conscientiousness 0.77).

We developed a Basque version of the OPERAS using a back-translation procedure and following the recommendations of International Test Commission (2018) regarding test translation and adaptation. The adequacy of the translated items with respect to the target population was examined through cognitive interviews and a pilot study. Specifically, cognitive interviews were used to analyze the complexity and the degree of understanding of the items, specific words, the instructions and the task to be carried out, and to question respondents about their answers, the purpose being to obtain validity evidence based on response processes (Caicedo and Zalazar-Jaime, 2018). Participants underwent a structured interview, answering individually. Participants in the pilot study were also asked about the complexity of the items and the terms used, and the extent to which they understood them. In this case, two open questions were added to the items of the scale. Participants in the cognitive interviews were 10 adults (5 women and 5 men) ranging in age from 41 to 63 years (*M* = 48.6; *SD* = 9.70), while the sample for the pilot study comprised 80 people (31 men and 49 women) ranging in age from 23 to 63 years (*M* = 46.01; *SD* = 7.72). All these participants were public sector employees resident in the Basque Country (northern Spain) and their proficiency in the Basque language ranged from upper intermediate to high (Levels B2 to C2 of the Common European Framework of Reference for Languages). The members of these samples did not participate in the empirical validation of the instrument. Based on suggestions made by participants in the cognitive interviews and in the pilot study, the team of translators reformulated items 27, 30, and 34 in order to facilitate their understanding. As part of the pilot study, item homogeneity indices were also calculated, and only item 13 yielded an index below 0.30. The wording of this item was consequently revised by the team of translators so as to improve its match to the corresponding theoretical dimension (*Openness to Experience*). The resulting instrument was the one used for the empirical validation of the Basque version of the OPERAS.

*Big Five Inventory (BFI; John et al., 1991; in its Spanish version, developed by Benet-Martínez and John, 1998)*. The BFI comprises 44 items, each rated on a 5-point Likert-type scale (from 1, “disagree strongly,” to 5, “agree strongly”), that assess the Big Five personality factors (Agreeableness, Openness to Experience, Neuroticism, Extraversion, and Conscientiousness). In the present sample, the internal consistency values were acceptable: Agreeableness 0.72; Openness to Experience 0.82; Neuroticism 0.83; Extraversion 0.86; and Conscientiousness 0.79. Some examples of the items are: “Likes to reflect, play with ideas” (Openness to Experience), “Is considerate and kind to almost everyone” (Agreeableness) or “Gets nervous easily” (Neuroticism).

*Piasentin’s (2007) questionnaire for assessing person-organization fit (in its Spanish version, developed by Cáceres, 2014).* The development of this instrument was based on the fact that the majority of research on PO Fit is oriented toward the measurement of supplementary adjustment (the similarity between the employees’ values and characteristics), from a direct-perceived perspective (employees themselves evaluate their adjustment to the organization) and focused on the values (Kristof-Brown et al., 2005). On the basis of these parameters Piasentin designed an instrument that presents good psychometric properties both in its original version and in the one adapted to Spanish (Cáceres, 2014). In the present sample, internal consistency (Cronbach’s alpha) was 0.70. The scale consists of 5 items rated using a 5-point Likert-type scale (from 1, “strongly disagree,” to 5, “strongly agree”). Some examples of the items are: “My coworkers and I share the same workplace ethics” and “My values match those of current employees in my organization.”

*Charbonnier-Voirin and Roussel’s (2012) scale for assessing adaptive performance (in its Spanish version, developed by Gorostiaga et al., in press)*. This 22-item scale measures five dimensions of adaptive performance (Managing Work Stress, Training Effort, Interpersonal Adaptability, Reactivity in the Face of Emergencies, and Creativity), with each item being rated on a 7-point Likert-type scale (from 1, “strongly disagree,” to 7, “strongly agree”). In the present sample internal consistency values (Cronbach’s alphas) were acceptable (Managing Work Stress 0.74, Training Effort 0.81, Interpersonal Adaptability 0.66, Reactivity in the Face of Emergencies 0.78, and Creativity 0.72). Some examples of the items are: “Within my department, people rely on me to suggest new solutions” (Creativity), “I willingly adapt my behavior whenever I need to in order to work well with others” (Interpersonal adaptability) or “I undergo training on a regular basis at or outside work to keep my competencies up to date” (Training Effort).

*Lee and Allen’s (2002) questionnaire for assessing OCBs (in its Spanish version, developed by Dávila and Finkelstein, 2010)*. This questionnaire comprises 16 items, each rated on a 5-point Likert-type scale (from 1, “never,” to 5, “always”), and it assesses both types of OCBs, that is, those directed at the organization (OCB-Os) and those directed at individuals (OCB-Is). The reliability coefficients (Cronbach’s alpha) in the present sample were 0.80 and 0.77 for these two dimensions, respectively. Some examples of the items are: “I demonstrate concern about the image of the organization” (OCB-O), “I give up time to help others who have work or non-work problems” (OCB-I).

### Procedure

Participants responded in person to the questionnaires in a single collective session in their respective workplaces under conditions that preserved anonymity. They were assisted by a researcher. The instruments were completed in the following order: Basque version of the OPERAS, Spanish version of the Big Five Inventory, Spanish version of the questionnaire for assessing OCBs, Spanish version of the questionnaire for measuring PO fit, and the Spanish version of the adaptive performance scale.

The study was approved by the Ethics Committee for Research Involving Humans of the University of the Basque Country.

### Data Analysis

For the validation of the Basque version of the OPERAS, we analyzed the following aspects:

(a) Items. We began by calculating descriptive statistics (mean, standard deviation, skewness, kurtosis) and the corrected homogeneity index. Following Curran et al. (1996), items with skewness and kurtosis values above 2 and 7, respectively, were considered problematic. In addition, and in accordance with item response theory (IRT), we estimated the item discrimination and threshold (difficulty) parameters (*a* and *b*, respectively).
(b) Dimensionality and construct validity. Here we performed a confirmatory factor analysis (CFA) and exploratory structural equation modeling (ESEM) using the weighted least squares (WLSMV) estimator and target rotation. Specifically, we conducted a CFA to analyze the fit of the data to the theoretical five-factor model of personality, with cross-loadings constrained to zero, and a less restrictive factor analysis in which cross-loadings were allowed. The Five-Factor Model (FFM) does not normally show adequate fit in CFAs because items are not pure indicators of a single factor and dimensions are usually correlated (Vassend and Skrondal, 2011). In addition, studies related to the FFM commonly use ESEM as it allows for testing of structures based on a prior theoretical model (Marsh et al., 2010; Booth and Hughes, 2014). In the present study, model fit was assessed using the following indices: the Tucker-Lewis index (TLI), the comparative fit index (CFI), and the root mean square error of approximation (RMSEA). For an acceptable fit, the rules of thumb are: TLI and CFI ≥ 0.90; RMSEA ≤ 0.08 (Hu and Bentler, 1999).
(c) Convergent validity. We calculated Pearson correlation coefficients between scores on the five dimensions of the OPERAS and scores on the BFI dimensions that measure the same constructs.
(d) Reliability. We calculated Cronbach’s alpha as internal consistency index. Furthermore, the test information function (TIF) was computed for each factor. The temporal stability of factors was examined using the test-retest technique, calculating Pearson correlation coefficients between scores obtained by a subsample of 151 participants over a 1-month interval.
(e) Differential item functioning with respect to participants’ first language (Basque or Spanish). Differential item functioning (DIF) was analyzed by applying logistic regression procedures (Swaminathan and Rogers, 1990) to each dimension of the OPERAS, using the specific syntax created by Slocum et al. (2004) for DIF detection in SPSS. Based on the criterion of Jodoin and Gierl (2001), an item is considered to show DIF when the effect size measure yields a value of Nagelkerke’s Δ*R*^2^ of at least 0.035.

Finally, and in order to address the objective of proposing an efficient model for selecting candidates most likely to perform well at work, we used hierarchical multiple regression models to examine the capacity of personality, PO fit, and adaptive performance to predict OCBs. As we have pointed out in the Introduction, these analyses contributed to provide evidence of validity based on relations to other variables, for the Basque adaptation of the scale.

The above analyses were performed using SPSS, Mplus, and IRTPRO.

## Results

We will begin by presenting the results for the validation of the Basque version of the OPERAS. Note that item 1 (dummy) was excluded from all analyses.

### Analysis of Instrument Items

Most of the items yielded mean scores around the midpoint of the scale and standard deviations close to 1. The means obtained were very similar to those reported for the original scale, with differences of no more than –0.5 or 0.5, except in the case of items 2, 22, 31, and 39. Differences in standard deviation were also small, between –0.2 and 0.2, except for items 22 and 33. All items, with the exception of items 10 and 13, yielded values of skewness and kurtosis below the threshold for problems of normality. Around 80% of items had a homogeneity index above 0.30. Those with a homogeneity index below this threshold were items 4, 5, 13, 18, 21, 28, 30, 35, and 38. Detailed data are presented in Table 1.

**TABLE 1:** Descriptive statistics and homogeneity indices for OPERAS items.

Item	Mean	Standard deviation	Skewness	Kurtosis	Homogeneity index
2. EX+	2.31	0.93	0.29	–0.42	0.50
3. ES+	4.04	0.65	–0.76	2.22	0.47
4. CO+	3.82	0.75	–0.72	1.44	0.19
5. SD−	4.29	0.62	–0.60	1.60	0.19
6. AG+	3.63	0.67	–0.59	0.75	0.40
7. OP−	2.00	0.99	0.74	–0.17	0.50
8. EX+	3.98	0.73	–0.89	2.13	0.50
9. ES−	1.88	0.92	1.12	1.05	0.56
10. CO−	1.54	0.87	2.23	5.73	0.37
11. SD+	2.62	1.40	0.33	–1.27	0.38
12. AG+	4.54	0.62	–1.73	6.21	0.38
13. OP+	4.59	0.71	–2.57	9.24	0.24
14. EX−	2.59	1.17	0.31	–0.86	0.54
15. ES−	1.84	0.85	1.08	1.27	0.57
16. CO−	1.85	0.87	1.18	1.56	0.44
17. AG+	3.77	0.76	–0.72	1.01	0.39
18. OP−	2.43	1.10	0.47	–0.56	0.24
19. SD+	3.50	1.16	–0.52	–0.67	0.38
20. EX+	3.69	0.94	–0.63	0.04	0.48
21. ES+	2.50	1.03	0.34	–0.62	0.20
22. CO−	1.97	0.97	1.06	0.87	0.40
23. AG−	2.50	0.96	0.24	–0.59	0.40
24. OP+	3.72	1.02	–0.58	–0.25	0.51
25. EX−	3.46	0.97	–0.34	–0.13	0.48
26. SD+	2.25	1.11	0.70	–0.38	0.45
27. ES−	1.93	0.95	0.93	0.26	0.40
28. CO+	3.71	0.98	–0.76	0.31	0.23
29. AG−	1.73	0.85	1.26	1.68	0.40
30. OP+	4.60	0.65	–1.75	3.35	0.27
31. EX−	3.54	1.07	–0.54	–0.17	0.44
32. ES−	2.15	0.96	0.76	0.11	0.47
33. CO−	2.03	0.92	0.81	0.33	0.43
34. AG+	3.94	0.79	–0.84	1.39	0.45
35. OP+	4.06	0.88	–1.24	1.97	0.22
36. EX+	3.48	0.78	–0.53	0.40	0.41
37. ES−	1.51	0.79	1.89	4.47	0.43
38. CO+	3.93	0.78	–1.27	3.17	0.28
39. AG−	2.06	0.93	0.71	0.19	0.40
40. OP−	2.08	1.12	0.84	–0.07	0.38

Parameter estimates based on the IRT graded response model (GRM) for the items of each scale dimension yielded discrimination indices ranging from 1.09 to 1.52 for Agreeableness, 0.57–2.29 for Openness to Experience, 0.49–2.51 for Emotional Stability, 1.02–1.87 for Extraversion, 0.55–1.92 for Conscientiousness, and 0.64–1.91 for Social Desirability. For the large majority of items, the value of the discrimination parameter (*a*) was above the threshold of 0.65 (Baker, 2001), the exceptions being items 18, 35, 21, 4, 28, and 5. However, all these items showed satisfactory fit to the GRM. The only items that showed poor fit to the GRM, *p* < 0.01, were items 13, 27, 10, and 38, although these items yielded adequate discrimination indices. Table 2 shows the data in full.

**TABLE 2:** Item parameter estimates using the graded response model for each of the six subscales of the OPERAS.

Item	Factor	*a*	*SE*	*b1*	*SE*	*b2*	*SE*	*b3*	*SE*	*b4*	*SE*	*X* ^2^	*df*	Prob.
6	Agreeableness	1.32	0.14	–4.79	0.59	–2.83	0.25	–0.53	0.08	2.75	0.25	30.24	35	0.69
12	Agreeableness	1.36	0.16	–4.29	0.50	–3.9	0.42	–3.38	0.34	–0.32	0.07	28.57	21	0.12
17	Agreeableness	1.09	0.12	–5.03	0.62	–2.99	0.29	–1.03	0.12	2.16	0.21	31.18	38	0.77
23	Agreeableness	1.09	0.11	–4.6	0.52	–1.81	0.18	–0.18	0.08	1.99	0.20	49.43	38	0.10
29	Agreeableness	1.24	0.13	–4.29	0.47	–2.97	0.28	–1.83	0.17	0.14	0.08	28.11	37	0.85
34	Agreeableness	1.52	0.15	–4.3	0.49	–2.45	0.20	–1.21	0.10	1.19	0.11	44.21	33	0.09
39	Agreeableness	1.16	0.12	–4.37	0.48	–2.57	0.24	–1.05	0.11	0.93	0.11	27.51	38	0.90
7	Openness	2.22	0.22	–2.91	0.23	–1.75	0.11	–0.74	0.07	0.36	0.06	35.10	38	0.60
13	Openness	0.94	0.12	–4.8	0.64	–4.62	0.60	–3.72	0.46	–0.9	0.13	58.20	32	0.00
18	Openness	0.57	0.09	–5.86	0.91	–2.76	0.42	–0.61	0.16	2.37	0.37	68.65	49	0.03
24	Openness	2.29	0.22	–2.51	0.18	–1.4	0.09	–0.47	0.06	0.90	0.08	52.50	40	0.09
30	Openness	0.79	0.12	–8.61	1.73	–5.81	0.88	–3.74	0.52	–1.07	0.17	30.72	31	0.48
35	Openness	0.62	0.09	–6.55	1.04	–4.61	0.69	–2.86	0.43	1.35	0.23	70.72	47	0.01
40	Openness	1.35	0.13	–3.08	0.27	–1.76	0.14	–0.92	0.09	0.54	0.09	58.94	46	0.09
3	Emotional stability	1.45	0.14	–4.73	0.61	–3.17	0.28	–1.71	0.14	1.33	0.12	49.18	36	0.07
9	Emotional stability	2.30	0.20	–2.85	0.21	–1.84	0.11	–1.16	0.08	0.35	0.06	50.61	37	0.07
15	Emotional stability	2.51	0.23	–2.97	0.23	–2.03	0.12	–1.21	0.08	0.35	0.06	59.37	36	0.01
21	Emotional stability	0.49	0.08	–3.55	0.61	0.47	0.18	3.00	0.53	7.86	1.40	66.91	52	0.08
27	Emotional stability	1.26	0.12	–4.34	0.46	–2.34	0.19	–1.37	0.12	0.45	0.09	87.72	44	0.00
32	Emotional stability	1.38	0.12	–3.76	0.35	–1.93	0.15	–0.97	0.09	1.04	0.11	48.38	42	0.23
37	Emotional stability	1.52	0.15	–3.53	0.33	–2.96	0.25	–2.11	0.17	–0.42	0.07	57.60	41	0.04
2	Extraversion	1.28	0.12	–1.36	0.13	0.32	0.08	2.20	0.19	4.21	0.43	39.67	46	0.73
8	Extraversion	1.87	0.18	–4.42	0.61	–2.42	0.18	–1.25	0.10	1.14	0.09	52.72	34	0.02
14	Extraversion	1.69	0.14	–2.30	0.17	–0.94	0.09	–0.07	0.07	1.22	0.10	58.08	47	0.13
20	Extraversion	1.71	0.15	–3.1	0.26	–1.64	0.12	–0.58	0.07	1.33	0.10	52.86	45	0.20
25	Extraversion	1.10	0.11	–2.05	0.19	0.03	0.09	1.83	0.18	4.08	0.42	59.11	51	0.20
31	Extraversion	1.02	0.11	–1.72	0.18	0.33	0.09	1.93	0.20	3.59	0.37	66.75	55	0.13
36	Extraversion	1.31	0.12	–3.92	0.38	–2.15	0.18	–0.14	0.08	2.65	0.22	58.26	47	0.17
4	Conscientiousness	0.63	0.10	–9.56	1.82	–5.05	0.78	–1.75	0.28	3.07	0.47	48.94	37	0.09
10	Conscientiousness	1.64	0.18	–2.79	0.25	–2.47	0.21	–2.13	0.17	–0.38	0.07	66.11	37	0.00
16	Conscientiousness	1.92	0.19	–3.11	0.28	–2.08	0.15	–1.43	0.10	0.41	0.07	56.54	36	0.02
22	Conscientiousness	1.44	0.14	–3.22	0.29	–2.13	0.17	–1.28	0.11	0.55	0.08	44.29	40	0.29
28	Conscientiousness	0.55	0.09	–7.17	1.24	–3.68	0.61	–1.43	0.26	2.81	0.47	57.81	48	0.16
33	Conscientiousness	1.61	0.15	–3.54	0.34	–2.07	0.16	–1.04	0.09	0.73	0.08	47.01	36	0.10
38	Conscientiousness	0.99	0.12	–4.81	0.58	–3.32	0.36	–1.84	0.19	1.80	0.20	79.37	40	0.00
5	Social desirability	0.64	0.11	–1.00	0.20	4.70	0.79	7.47	1.36	9.45	1.92	38.73	25	0.04
11	Social desirability	1.16	0.15	–0.98	0.13	0.20	0.09	–0.75	0.11	2.10	0.22	41.23	33	0.15
19	Social desirability	1.19	0.15	–2.82	0.30	–1.26	0.14	–0.38	0.09	1.46	0.16	53.32	30	0.01
26	Social desirability	1.91	0.30	–0.80	0.09	0.59	0.08	1.24	0.12	2.56	0.24	48.32	28	0.01

*a, discrimination parameter; b1, b2, etc., threshold parameters; SE, standard error; df, degrees of freedom.*

For most of the scale dimensions, the TIF curves peaked between –2 and 1 on their underlying construct axis, suggesting that scores on the Basque version of the OPERAS are more precise toward the lower end or middle of the measurement scale (theta < 1). The TIF curves are shown in Supplementary Figure 1.

### Dimensionality and Construct Validity

Although the fit of the CFA model was not good [χ^2^(687) = 2476.80, *p* < 0.001; RMSEA = 0.06; CFI = 0.80; TLI = 0.79], the results of ESEM indicated acceptable fit [χ^2^(522) = 1131.81, *p* < 0.001; RMSEA = 0.04; CFI = 0.93; TLI = 0.91]. In addition, and as can be seen in Table 3, all items except items 4 and 5 had a loading above 0.30 on their target factor, and cross-loadings were below this value on all except items 5, 6, 23, and 31.

**TABLE 3:** Standardized factor loadings from the ESEM for the five-factor model.

Item no.	Agreeableness	Openness to experience	Emotional stability	Extraversion	Conscientiousness	Social desirability
6	**0.56**	0.02	–0.02	–0.02	0.04	–0.30
12	**0.46**	0.13	–0.01	–0.04	0.22	–0.13
17	**0.53**	–0.01	0.08	0.03	–0.16	0.03
23	**0.56**	–0.04	0.27	–0.04	–0.30	–0.13
29	**0.49**	0.02	0.14	0.09	0.12	0.07
34	**0.56**	0.08	0.16	–0.04	–0.03	–0.10
39	**0.44**	0.05	0.03	0.06	0.07	0.01
7	–0.17	**0.82**	0.09	–0.05	–0.11	–0.07
13	0.01	**0.49**	0.04	–0.10	0.06	0.18
18	–0.02	**0.34**	–0.01	0.05	0.01	0.06
24	–0.11	**0.80**	0.08	0.00	–0.15	–0.10
30	0.21	**0.35**	–0.03	0.09	0.15	0.10
35	0.13	**0.31**	–0.02	0.02	0.16	0.14
40	0.14	**0.53**	–0.11	0.11	–0.08	–0.14
3	0.12	0.06	**0.49**	0.02	0.15	0.00
9	–0.06	–0.01	**0.81**	–0.04	0.11	0.10
15	0.00	–0.02	**0.78**	0.03	0.05	0.09
21	–0.01	–0.03	**0.30**	0.10	–0.15	–0.22
27	0.13	0.01	**0.44**	0.12	0.06	0.00
32	0.21	0.10	**0.49**	–0.03	0.07	–0.07
37	0.17	0.13	**0.50**	0.05	0.14	0.16
2	–0.18	0.05	–0.02	**0.62**	–0.08	0.01
8	0.27	–0.01	0.00	**0.67**	0.06	0.16
14	–0.11	0.01	0.00	**0.67**	0.03	0.10
20	0.28	0.01	–0.09	**0.65**	0.09	0.13
25	–0.27	–0.04	0.09	**0.64**	–0.11	–0.25
31	–0.23	–0.01	0.12	**0.60**	–0.17	–0.33
36	0.11	0.05	–0.04	**0.54**	0.20	0.18
4	0.05	0.17	0.07	0.09	**0.22**	0.06
10	0.07	0.02	0.18	–0.03	**0.54**	–0.09
16	–0.06	–0.11	0.19	–0.03	**0.65**	–0.08
22	–0.11	0.00	0.24	–0.03	**0.52**	–0.11
28	–0.14	0.15	–0.22	–0.05	**0.48**	–0.09
33	0.01	–0.03	0.17	0.11	**0.55**	–0.13
38	0.11	0.03	0.05	0.11	**0.41**	0.02
5	–0.11	–0.09	–0.03	0.06	–0.43	**0.10**
11	–0.06	–0.03	0.08	–0.05	–0.18	**0.44**
19	–0.26	0.03	0.06	0.01	–0.03	**0.69**
26	–0.21	–0.13	0.08	–0.02	–0.25	**0.45**

*Values in bold are loadings on the target factor.*

### Convergent Validity

As can be seen in Table 4, Pearson correlation coefficients between scores on the OPERAS dimensions and those on the BFI ranged from 0.62 to 0.77. As expected, scores on Neuroticism (BFI) showed a significant inverse association of large magnitude (–0.71) with scores on the Emotional Stability dimension of the OPERAS. The association between scores on the Conscientiousness dimension of the two measures (BFI and OPERAS) was also high, and in this case positive (0.68). Scores on the remaining OPERAS dimensions showed high positive correlations with scores on their corresponding BFI dimension. The median correlation coefficient obtained was 0.68, which can be considered excellent (Muñiz et al., 2011).

**TABLE 4:** Bivariate correlations between OPERAS and BFI dimensions.

OPERAS	BFI	r
Agreeableness	Agreeableness	0.68
Openness to experience	Openness to experience	0.62
Emotional stability	Neuroticism	–0.71
Extraversion	Extraversion	0.77
Conscientiousness	Conscientiousness	0.68

### Reliability

There was good support for reliability with Cronbach’s alpha values ranging from 0.62 to 0.76. Specifically, the values were 0.70 for Agreeableness; 0.62 for Openness to Experience; 0.72 for Emotional Stability; 0.76 for Extraversion; and 0.62 for Conscientiousness.

Regarding temporal stability, correlation coefficients between factor scores over a 1-month interval ranged from 0.62 (Agreeableness) to 0.85 (Extraversion). The median value of the test-retest reliability coefficient for the Big Five was 0.67, which can be regarded as adequate (Muñiz et al., 2011).

### Differential Item Functioning With Respect to Participants’ First Language (Basque or Spanish)

Table 5 shows the results of the analyses conducted to examine whether the respondent’s first language had an impact on item functioning. It can be seen that the value of Nagelkerke’s Δ*R*^2^ was only above 0.035 in the case of two items: 9 and 21. Both these items measure Emotional Stability and they showed uniform DIF, with higher scores among respondents with Basque as their first language on item 9 and among respondents with Spanish as their first language on item 21. The effect size observed, below 0.07, indicates that these two items showed moderate DIF.

**TABLE 5:** Differential item functioning of OPERAS items with respect to participants’ first language (Basque or Spanish).

Item	Total DIF Δ*R*^2^ Nagelkerke	Uniform DIF Δ*R*^2^ Nagelkerke	Non-uniform DIF Δ*R*^2^ Nagelkerke
2. EX+	0.00	0.00	0.00
3. ES+	0.01	0.01	0.01
4. CO+	0.00	0.00	0.00
5. SD−	0.01	0.00	0.01
6. AG+	0.01	0.01	0.00
7. OP−	0.00	–0.01	0.00
8. EX+	0.01	0.01	0.00
9. ES−	0.05	0.04	0.00
10. CO−	0.00	0.00	0.00
11. SD+	0.00	0.00	0.00
12. AG+	0.01	0.01	0.00
13. OP+	–0.01	–0.01	0.00
14. EX−	0.00	0.00	0.00
15. ES−	0.02	0.01	0.01
16. CO−	0.01	0.00	0.00
17. AG+	0.02	0.00	0.01
18. OP−	0.00	0.00	0.00
19. SD+	0.03	0.03	0.00
20. EX+	0.00	0.00	0.00
21. ES+	0.06	0.06	0.00
22. CO−	0.00	–0.01	0.00
23. AG−	0.01	0.00	0.01
24. OP+	0.01	0.01	0.00
25. EX−	0.02	0.01	0.01
26. SD+	0.00	0.00	0.00
27. ES−	0.00	0.00	0.00
28. RE+	0.01	0.00	0.00
29. AG−	0.01	0.00	0.01
30. OP+	0.01	0.01	0.01
31. EX−	0.02	0.02	0.00
32. ES−	0.00	0.00	0.00
33. CO−	0.01	0.00	0.01
34. AG+	0.01	0.01	0.00
35. OP+	0.03	0.02	0.00
36. EX+	0.01	0.00	0.00
37. ES−	0.00	0.00	0.00
38. CO+	0.01	0.00	0.00
39. AG−	0.00	0.00	0.00
40. OP−	0.02	0.02	0.00

*AG, Agreeableness; OP, Openness to Experience; ES, Emotional Stability; EX, Extraversion; CO, Conscientiousness; SD, Social Desirability. The “+” and “–” symbols indicate positive and negatively worded items, respectively.*

In the next section, we present the results regarding our proposed model for enhancing the efficiency of personnel selection.

### Capacity of Personality, Person-Organization Fit, and Adaptive Performance to Predict Organizational Citizenship Behaviors

Table 6 shows the results of the hierarchical multiple regression with OCB-O as the criterion variable. It can be seen that personality explained 15% of the variance in OCBs directed at the organization. When PO fit was added to the model, the adjusted *R*^2^ increased from 0.15 to 0.18, which implies an additional 20% of explained variance than was accounted for by personality alone. The further addition of adaptive performance produced a model that explained 40% of the variance in OCB-O, an increase of 112.22% over the variance that was accounted for by the combination of personality and PO fit.

**TABLE 6:** Hierarchical multiple regression with OCB-O as the criterion variable.

Model	Variables	*R*	Adjusted *R*^2^	Δ*R*^2^	Change in *F*
1	Personality	0.40	0.15		20.44***
2	Personality, PO fit	0.43	0.18	0.03	19.85***
3	Personality, PO fit, Adaptive performance	0.64	0.40	0.22	48.47***

***p < 0.001.

Table 7 shows the results of the hierarchical multiple regression with OCB-I as the criterion variable. It can be seen that personality explained 15% of the variance in OCBs directed at the individual. With the addition of PO fit to the model, the adjusted *R*^2^ increased to 0.19, which implies an additional 26.67% of explained variance than was accounted for by personality alone. The further addition of adaptive performance produced a model that explained 31% of the variance in OCB-I, an increase of 63.16% over the variance that was accounted for by the combination of personality and PO fit.

**TABLE 7:** Hierarchical multiple regression with OCB-I as the criterion variable.

Model	Variables	*R*	Adjusted *R*^2^	Δ *R ^2^*	Change in *F*
1	Personality	0.40	0.15		20.35***
2	Personality, PO fit	0.44	0.19	0.04	27.56***
3	Personality, PO fit, Adaptive performance	0.57	0.31	0.11	24.24***

***p < 0.001.

## Discussion

The primary aim of this study was to develop and validate a Basque version of the OPERAS, a scale designed to assess the Big Five personality factors and which is applicable to a wide range of settings. Having done so, we then aimed to propose an efficient model for selecting the candidates most likely to perform well at work, by examining whether the capacity of personality to predict OCBs was enhanced by the inclusion of PO fit and adaptive performance in the predictive model.

Regarding the Basque version of the OPERAS, the descriptive statistics, homogeneity indices, and the ICCs indicate that the majority of scale items have adequate psychometric properties. In addition, the results of the factor analysis confirm that the internal structure of the adapted scale is consistent with the Big Five model of personality. As in other studies (Marsh et al., 2010; Vassend and Skrondal, 2011; Chiorri et al., 2016), we found that the model in which some cross-loadings were allowed showed a better fit than did the independent cluster model of CFA, indicating that the less restrictive model more closely reflects the nature of questionnaires used to assess personality.

With respect to convergent validity, the median correlation between scores on the OPERAS dimensions and those of the BFI that measure the same construct (0.68) was very similar to that reported in the original validation study (0.69), and it may be considered excellent. As regards differential validity (analysis of DIF), only two items showed DIF in relation to the respondent’s first language and they account for just 5% of the total number of test items, much lower than the figure of around 20% of items with DIF that is often observed with adapted tests (Hidalgo-Montesinos et al., 2015). Furthermore, as one of them benefited respondents with Basque as their first language while the other benefited people who had Spanish as their first language, they may operate in opposing directions and balance each other out. On the other hand, as both belonged to the same dimension, deleting them would worsen the test’s construct validity. Also, considering that the instrument has been adapted from another language and culture, we believe that their removing would preclude cross-cultural comparisons. For these reasons, we decided to keep these two items in the test.

Finally, and concerning the instrument’s reliability, the indices of internal consistency (based both on classical test theory and IRT) and temporal stability were all acceptable. Although the median test-retest reliability coefficient for the Basque version (0.67) was lower than that reported for the original scale (0.73), this difference may be due to the fact that our sample was smaller and more homogeneous than that used by the scale authors. Also noteworthy is that the test information functions of scale factors indicated greater precision at lower or intermediate levels of the measured construct, suggesting that the Basque version of the OPERAS is a suitable instrument for identifying candidates whose personality profile is not ideal for a given post.

In summary, the validity evidence obtained in the process of adapting the OPERAS indicates that it is a valid and reliable instrument for assessing personality, based on the Big Five model, in the Basque-speaking population.

Regarding the second study objective of proposing an efficient model for selecting candidates most likely to perform well at work, the results indicate that the model combining personality, PO fit, and adaptive performance offers adequate prediction of OCBs. The addition of PO fit increased the capacity of personality to predict OCB-I, with the effect size being slightly higher than that reported by Tsai et al. (2012) and lower than that obtained by Michaud (2014). However, the latter difference may be due to the different instruments used in the two studies, insofar as Michaud used an overall measure of OCBs, an ipsative personality test, and an indirect procedure for measuring PO fit. Our finding that PO fit added incremental validity to personality in the prediction of OCB-I is consistent with the results obtained by Wei (2012), who found that employees with high levels of PO fit engaged in more OCB-Is and saw these behaviors as an investment that would be rewarded with more support from work colleagues. In our study, PO fit also increased the capacity of personality to predict OCB-Os, suggesting that a better fit to the organization will be reflected in behaviors aimed at improving it, such as loyalty, putting forward ideas for better organizational functioning, or consistently abiding by organizational rules and policies. Given that PO fit is not influenced by sociodemographic and employment variables such as gender, age, level of education or work experience (Tsai et al., 2012; Xu, 2014), it would appear to be a highly useful construct in the context of personnel selection.

Our analysis also showed that the addition of adaptive performance to the model comprising personality and PO fit improved notably its predictive capacity. Although the prediction of both types of OCBs was improved by the inclusion of adaptive performance in the model, the effect was greater with respect to OCB-Os. Importantly, the combined predictive capacity in this latter case was as high as that achieved with the best combinations of three predictors of job performance (Salgado, 2017). Furthermore, this combined predictive capacity is greater than that obtained by Murphy (2015) when using individual adaptability, Conscientiousness (as a personality factor), and cognitive ability as predictors of OCBs. The results linked to the second goal of the study help to provide evidence of validity based on relations to other variables, for the Basque adaptation of the instrument.

Finally, it should be noted that in addition to the practical contributions of the present study, the results obtained support the I-ADAPT model of Ployhart and Bliese (2006), one that provides a broad theoretical basis for proposing and testing empirically further models aimed at identifying those candidates most likely to perform well at work.

### Limitations

The data were obtained through a cross-sectional research design and in a non-selective context. This may have modified the size of the correlations between 0.05 and 0.10 compared to those obtained through predictive validity designs in selection contexts. In addition, participants were public sector workers, and so the results cannot be generalized to the private sector context. Given that instrument validation is an ongoing process, it would be advisable to gather further validity evidence from samples involving employees both from the private sector and of different ages to those included here. This, in turn, would help to increase the external validity of our proposed predictive model. A further limitation to consider is that personality assessment was based solely on self-reports, which according to Sackett and Lievens (2008) should be complemented with other approaches (interviews, informant ratings). It should be noted, however, that the information provided by self-report measures is reliable and allows valid inferences with regard to personality (Clark and Watson, 2019). Furthermore, recent studies (Fronczyk, 2019; Mõttus et al., 2020) support the measurement invariance of personality constructs across both self-reports and informant ratings. In the same vein, the studies by Demerouti et al. (2014), as well as the meta-analysis by Carpenter et al. (2014), indicate that self-appraisals may be the best way to measure OCBs. Nevertheless, appraisals by coworkers or managers would have provided a complementary perspective.

## Conclusion

As regards the Basque version of the OPERAS, whose validation was the main goal of this study, its psychometric properties indicate that it is a suitable instrument for assessing personality in Basque public sector employees. Given that it is a brief instrument that controls for social desirability and acquiescence effects, its availability not only helps to safeguard the right of Basque speakers to be assessed in their first language but also provides a useful tool for selection processes in which personality is assessed alongside other constructs with the aim of predicting job performance.

In fact, the results obtained show that consideration of PO fit and adaptive performance can notably improve the capacity of personality to predict OCBs. Furthermore, the predictive capacity achieved in relation to OCB-Os was as high as that obtained with the best combinations of three predictors of job performance. A practical implication of the proposed model is that it suggests the possibility of achieving a highly valid selection design by administering short instruments that can be completed in just over an hour. Hence, the new predictive model we propose may enhance the efficiency of personnel selection processes.

## Data Availability Statement

The raw data supporting the conclusions of this article will be made available by the authors, without undue reservation.

## Ethics Statement

The studies involving human participants were reviewed and approved by the Ethics Committee for Research Involving Humans of the University of the Basque Country. The patients/participants provided their written informed consent to participate in this study.

## Author Contributions

NB: conceptualization, funding acquisition, project administration, and writing the original draft. AG: conceptualization, data curation, and formal analysis. AR-L: investigation and methodology. JA: investigation, visualization, and writing—review and editing. All authors contributed to the article and approved the submitted version.

## Conflict of Interest

The authors declare that the research was conducted in the absence of any commercial or financial relationships that could be construed as a potential conflict of interest.

## Publisher’s Note

All claims expressed in this article are solely those of the authors and do not necessarily represent those of their affiliated organizations, or those of the publisher, the editors and the reviewers. Any product that may be evaluated in this article, or claim that may be made by its manufacturer, is not guaranteed or endorsed by the publisher.
