# Supplementary figures and images for: Assessment of Personality in Basque Public Sector Employees and Its Role in Predicting Organizational Citizenship Behaviors in Selection Processes
Source: Front Psychol. 2021 Dec 10;12:787850. doi: 10.3389/fpsyg.2021.787850 (PMC8702496; doi:10.3389/fpsyg.2021.787850)

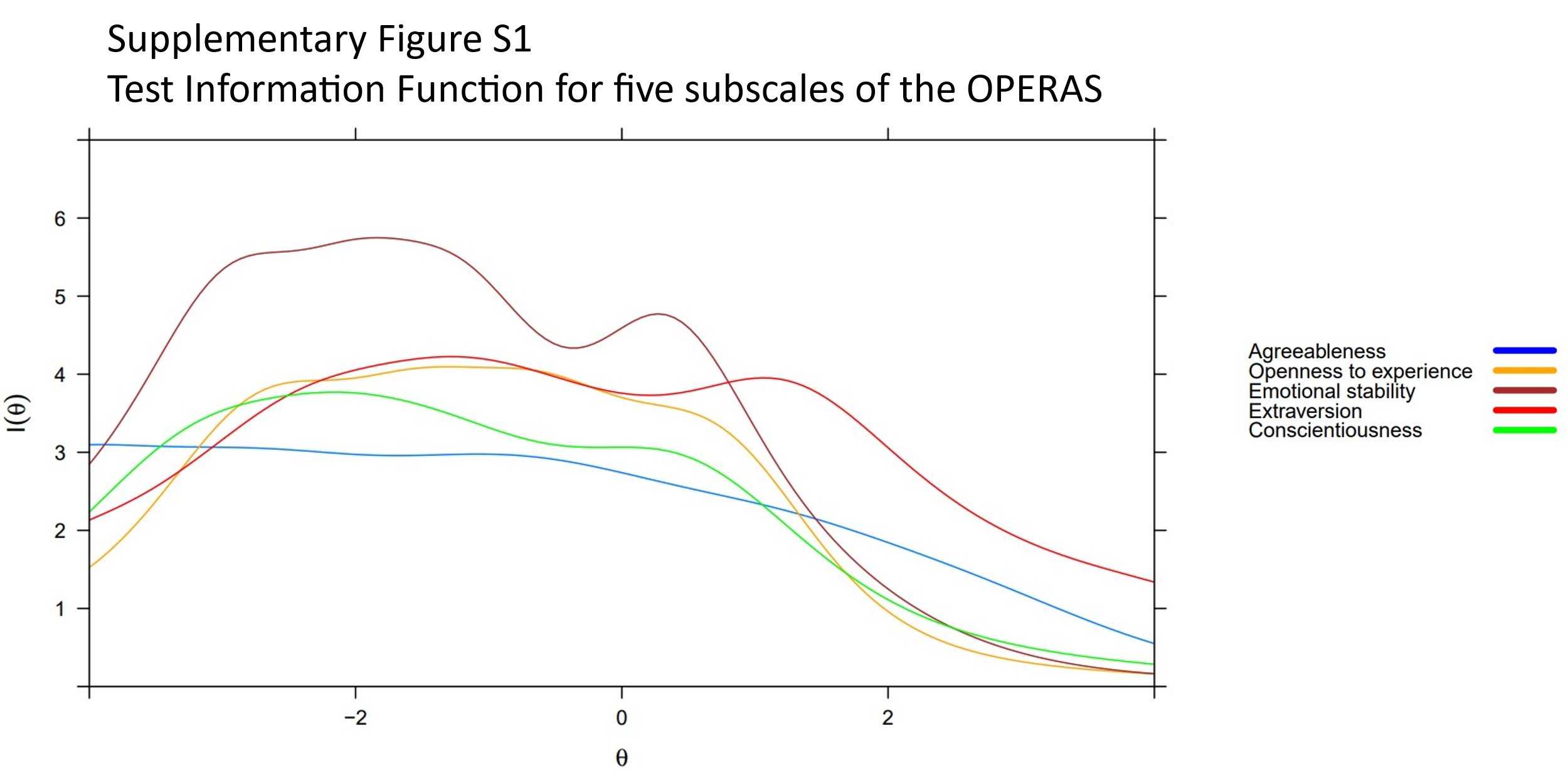

Supplement: Supplementary file 1 [file Table_1.DOCX]
